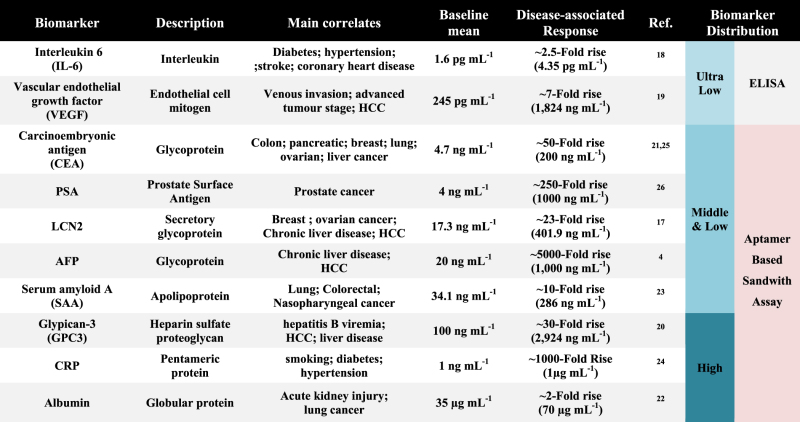# Aptamer-based Sandwich Assay and its Clinical Outlooks for Detecting Lipocalin-2 in Hepatocellular Carcinoma (HCC)

**DOI:** 10.1038/srep10897

**Published:** 2015-06-03

**Authors:** Kyeong-Ah Lee, Ji-Young Ahn, Sang-Hee Lee, Simranjeet Singh Sekhon, Dae-Ghon Kim, Jiho Min, Yang-Hoon Kim

**Affiliations:** 1 Department of Microbiology, Chungbuk National University, 1 Chungdae-Ro, Seowon-Gu, Cheongju 362-763, South Korea; 2 Division of Gastroenterology and Hepatology, Research Institute of Clinical Medicine, Department of Internal Medicine, Chonbuk National University, Medical School and Hospital, Jeonju, 561-756, South Korea; 3 Graduate School of Semiconductor and Chemical Engineering, Chonbuk National University, 567 Baekje-daero, Deokjin-Gu, Jeonju, 561-756, South Korea

## Abstract

We validated a single-stranded, DNA aptamer-based, diagnostic method capable of detecting Lipocalin-2 (LCN2), a biomarker from clinically relevant hepatocellular carcinoma (HCC) patient serum, in the sandwich assay format. Nine aptamers (LCN2_apta1 to LCN2_apta9) for LCN2 were screened with SELEX processes, and a sandwich pair (LCN2_apta2 and LCN2_apta4) was finally chosen using surface plasmon resonance (SPR) and dot blotting analysis. The result of the proposed aptamer sandwich construction shows that LCN2 was sensitively detected in the concentration range of 2.5–500 ng mL^−1^ with a limit of detection of 0.6 ng mL^−1^. Quantitative measurement tests in HCC patients were run on straight serum and were compared with the performance of the conventional antibody-based ELISA kit. The aptamer sandwich assay demonstrated an excellent dynamic range for LCN2 at clinically relevant serum levels, covering sub-nanogram per mL concentrations. The new approach offers a simple and robust method for detecting serum biomarkers that have low and moderate abundance. It consists of functionalization, hybridization and signal read-out, and no dilution is required. The results of the study demonstrate the capability of the aptamer sandwich assay platform for diagnosing HCC and its potential applicability to the point-of-care testing (POCT) system.

Hepatocellular carcinoma (HCC) is responsible for 5% of all deaths worldwide^1^. Liver disease such as cirrhosis and HCC is the 5^th^ most common cancer, after thyroid, stomach, colon and lung cancer, in Asian countries^2^. According to the mortality report from the Korean Statistical Information Service (KOSIS, http://kosis.kr), HCC deaths in Korea gradually increased an average of 5 percent annually from 2003 to 2011 (STATISTICS Korea, http://kostat.go.kr). This increase can be attributed to a rise in the incidence of viral hepatitis infection—hepatitis B virus (HBV) and hepatitis C virus (HBC)—and cirrhosis^3^. Despite the advances in therapeutic techniques and diagnostics in HCC, the fatality rate for liver disease remains very high because most of the patients are diagnosed at an advanced or late stage^2^.

With respect to the diagnostic investigation of HCC, blood tests (serum biomarkers), imaging and histological confirmation have been standard^4^,^5^. However, because of the drawbacks of liver biopsies, such as incorrect targeting and potential tumor-cell seeding, biomarkers and imaging studies are more commonly used for HCC diagnosis^4^,^6^. Numerous serum disease markers for diagnosing HCC have been developed, including α-fetoprotein (AFP), des-γ-carboxy prothrombin (DCP), protein induced by a lack of vitamin K or antagonist (PIVKA-II) and a fucosylated variant of the AFP glycoprotein (AFP-L3)^7^,^8^,^9^. In particular, AFP has been intensively studied for diagnosing HCC patients, with a cutoff value of 20 ng mL^−1^^4^. A few studies, however, have indicated that the use of AFP as single biomarker for HCC has limitations because of its variability in specificity and sensitivity depending on the assay factors, such as the experimental platform design, sample size and volume^10^,^11^. To overcome the limitations of the use of only a single or a few biomarkers, additional innovative biomarkers have been developed that improve diagnostic discrimination in HCC^12^,^13^.

LCN2 (Lipocalin-2; neutrophil gelatinase-associated lipocalin (NGAL)), a 24 kDa secretory glycoprotein that was first identified in urine collected from mice with SV40-infected kidneys, is stored in human neutrophils^14^,^15^. The primary function of LCN2 is thought to be related to the transport of small ligands, which have been implicated in inflammation, iron metabolism, and the induction of apoptosis^16^. Recently, high expression of LCN2 was observed in a HCC-microarray analysis study, suggesting the potential for LCN2 to be a quantitative biomarker^13^. In serum, the LCN2 concentration in patients with chronic liver disease was higher than that of healthy individuals (median 67.45 ng mL^−1^ (range 17.3–401.9 ng mL^−1^) vs. 57.9 ng mL^−1^ (range 18.3–176.3 ng mL^−1^))^17^. We assumed that including LCN2 detection and quantitation in multiple biomarker sets might assist with the diagnosis, prognosis and therapy monitoring of HCC progress.

Biomarkers can be indicative a variety of disease characteristics, and they are strongly correlated with disease progression. Many studies of disease biomarkers have reported elevated levels in various cancers^4^,^17^,^18^,^19^,^20^,^21^,^22^,^23^,^24^,^25^. For example, normal levels of prostate-specific antigen (PSA) are typically 0.5–2 ng mL^−1^ 26. A PSA serum concentration of 4 to 10 ng mL^−1^ indicates the possibility of early-stage prostate cancer. The late stage is characterized by elevated values of 10 to 1000 ng mL^−1^. Therefore, it is necessary to select an appropriate diagnostic method according to the disease characteristics and purpose of use, such as for early diagnosis, prognosis and monitoring. In fact, most previous biomedical studies have focused primarily on measuring ultra-low or low abundance serum markers based on the enzyme-linked immunosorbent assay (ELISA) platform because it is a highly sensitive detection method, and the use of commercial antibodies is attractive^27^. However, this serum use is problematic due to the highly non-specific interaction and signal interference from the most abundant serum proteins, which generate a non-measurable signal. Therefore, pre-treatment, such as long-turnaround serial dilution, is always required to obtain reliable results in clinical tests^28^. There is an urgent need to develop a diagnostic strategy that satisfies both the linearity in the range of sub-nanograms and high simplicity for clinical sample use^29^.

Here we highlight the use of aptamer-based sandwich assay technology. Aptamers are functional, single stranded oligonucleotides with high affinity for various targets, such as proteins, small molecular, cells, and microorganisms^30^. Various intramolecular interactions among the components in aptamer sequences lead to the formation of a large variety of secondary or tertiary structures once they properly fold, allowing them to recognize and bind to a specific target^31^. They can be generated using an iterative approach, called systematic evolution of ligands by exponential Enrichment (SELEX). Aptamers offer assay stability, easy regeneration (polymerase chain reaction (PCR) or chemical synthesis) and simple modification for detecting the target of interest in direct, indirect and sandwich concepts^32^. We validated the use of the aptamer-based sandwich assay for LCN2, which contains two major elements: a biological recognition element that captures LCN2 (Capture_Aptamer) and a reporter element that converts the target binding into a detectable signal-readout (Reporter_Aptamer). The data presented here demonstrate the feasibility of using the aptamer-based sandwich assay to detect LCN2 in very high serum concentrations. The results obtained with clinical samples are comparable to those with the commercial ELISA kit. Moreover, our platform accomplishes the direct detection of biomarker proteins within low to moderate abundance (ng–μg mL^−1^)^33^,^34^. LCN2 detection by the aptamer assay offers many additional advantages, such as on-site detection, minimization of assay steps, and cost-effectiveness, which make it an ideal approach to meet the growing need for improved point-of-care testing (POCT) HCC diagnosis.

## Materials and Methods

### Ethics statement

Clinical samples were collected from the Institute of Clinical Medicine, Department of Internal Medicine, Chonbuk National University Hospital, South Korea. All 8 blood/serum samples (from 4 normal individuals and 4 clinically relevant HCC patients) were collected in accordance with the approved guidelines and relevant regulations. All subjects provided their written informed consent prior to participating in the study. The Ethics Committee and IRB (Institutional Review Board) of Chonbuk National University Hospital approved all experimental procedures. All experiments were performed in accordance with the approved guidelines.

### Proteins and buffer solutions

C-terminal polyhistidine-tagged recombinant human Lipocalin-2 (LCN2) was purchased from Sino Biological Inc. (Beijing, China). Anti-Lipocalin-2 monoclonal antibody was purchased from Abcam (Cambridge, UK), and anti-mouse IgG peroxidase conjugate was purchased from Sigma-Aldrich Inc. (St. Louis, MO, USA). Glutathione S-transferase (GST), bovine serum albumin (BSA) and α-fetoprotein (AFP) were purchased from Sino Biological Inc. and Sigma-Aldrich Inc. Human serum albumin (HSA) was purchased from MyBioSource (San Diego, CA, USA). Solutions for the SELEX experiment were prepared as follows: binding buffer (500 mM NaCl, 20 mM Tris-HCl, 5 mM imidazole, pH 7.9), washing buffer (500 mM NaCl, 20 mM Tris-HCl, 60 mM imidazole, pH 7.9), and elution buffer (300 mM NaCl, 20 mM Tris-HCl, 250 mM imidazole, pH 7.9). Phosphate-buffered saline (PBS) buffer (pH 7.2) was purchased from Gibco BRL (New York, USA). For the dot blotting, PBS-T buffer (0.1% v/v Tween 20 in PBS, pH 7.2) and washing buffer (0.5% v/v Tween 20 in PBS, pH 7.2) were prepared.

### *In vitro* selection of aptamers by SELEX

DNA aptamer pools were prepared using a previously described method with some modifications^30^. DNA templates contained a central 40 random nucleotides flanked by an invariant 18-nucleotide region (5’-ATACCAGCTTATTCAATT (N40) AGATAGTAAGTGCAATCT-3’). The pool was amplified through PCR and then purified with a PCR purification kit (Qiagen, Korea). The initial ssDNA pools (742 pmol) were denatured at 95 °C for 5 min in 100 μL of binding buffer and slowly cooled to room temperature for the intrinsic 3-D structure formation. DNA aptamers were then mixed with human LCN2 protein (74.2 pmol) and incubated for 1 h at 4 °C. The LCN2-aptamer complexes were bound to Ni-NTA agarose (Qiagen; pre-equilibrated with binding buffer) at 4 °C for 1 h. After washing, LCN2-aptamer complexes were eluted with elution buffer. LCN2-bound aptamers were treated with PCI (phenol/chloroform/isoamyl alcohol, 25:24:1, v/v) and sequentially precipitated by ethanol at −20 °C. The aptamer sub-pools were collected and directly diluted in 100 μL of Milli-Q water. The binding recoveries during SELEX was compared with each other as described in the **Supporting Information** (Table S1 and Figure S1).

Aptamer sub-pools of LCN2 were amplified by PCR for the next round of selection. The amplification condition for PCR was as follows: an initial denaturation at 95 °C for 1 min followed by 20 amplification cycles of 45 s of denaturation at 94 °C, 1 min annealing at 55 °C, 1 min elongation at 72 °C and final elongation of 5 min at 72 °C. ssDNA for the subsequent SELEX rounds was prepared as previously described^30^,^35^. After selecting the 6^th^ round in SELEX, the LCN2-specific binding DNA aptamer was recovered by affinity techniques with Ni-NTA agarose without LCN2 protein, which was the negative round (N.R) of SELEX. After ten selection rounds, the eluted aptamer concentrations at each round were assessed using a NanoDrop spectrophotometer (Thermo Scientific, Rochester, NY, USA). The sequences were aligned using the ClustalX software (version 1.83).

### Post-SELEX and real-time PCR analysis

To select the optimal selection round, post-SELEX was followed by the SELEX protocol as described above. The eluted ssDNA concentrations from each round in SELEX were quantified by measuring the absorbance at 260 nm using a NanoDrop spectrophotometer (Thermo Scientific, USA). The binding DNA aptamer pools from the initial round and the 7^th^, 8^th^, and 9^th^ rounds were prepared at same concentration (742 pmol) and incubated with human LCN2 protein (74.2 pmol) in parallel for 1 h. After three washes, bound ssDNA was retrieved from the cell-pool complexes. Serial dilutions of each sample (1 × –no dilution, 10 × ) were prepared and analyzed through real-time PCR using a MiniOpticon^TM^ real-time PCR fluorescence signal detection system (Bio-Rad, USA). For the amplification, 20 μL reaction mixtures were prepared, consisting of 10 μL of 1X iQ SYBR Green Supermix (Bio-Rad, USA), 8.8 μL of water, 0.1 μL of 0.1 mM of forward primer, 0.1 μL of 0.1 mM of reverse primer, and ten-fold serial dilution of each template DNA (1 μL). The PCR parameters consisted of the following: Taq activation (3 min at 94 °C); 40 cycles of PCR at 94 °C for 30 sec, 52 °C for 30 sec, and 72 °C for 15 sec; and 5 min of extension at 72 °C. All sample analyses were performed in triplicate. For analysis, the efficiency measurement and threshold cycle (Ct) analysis were conducted with version 3.1 of the MJ Opticon Monitor analysis software (Bio-Rad, USA).

### Affinity measurement of the LCN2-bound aptamer using surface plasmon resonance (SPR)

The binding affinities of the isolated aptamers (42 clones) to LCN-2 were analyzed by Surface Plasmon Resonance using a BIAcore 3000 Instrument (GE Healthcare, Sweden) at 24 °C. We used the CM5 chip (GE Healthcare, Sweden), a carboxymethylated 3D dextran matrix, which is a general purpose chip for immobilizing a wide range of ligands, such as proteins, nucleic acids and carbohydrates. The CM5 chip, immobilized with LCN2, was investigated in terms of the aptamer affinity (K_*D*_) using the BIAcore 3000. Immobilization of LCN2 on a CM5 chip (GE Healthcare, Uppsala, Sweden) was performed according to the standard protocol of the amine-coupling method (BIAapplication Handbook, BIAcore® AB) (**Supporting information** Figure S3). Before LCN2 immobilization, freshly prepared 0.4 M N-(3-dimethylaminopropyl)-N-ethylcarbodiimide hydrochloride (EDC) and 0.1 M N-hydroxysulfosuccinimide (sulfo-NHS) were mixed in a 1:1 ratio and passed over the sensor chip for 10 min, followed by 60 μg mL^−1^ LCN2 in 10 mM sodium acetate at pH 4.5 for 6 min. Unreacted carboxyl groups were then quenched with 1 M ethanolamine at pH 8.5 for 10 min.

For the cross-reactivity test, streptavidin immobilized sensor chip SA (GE, for BIAcore 3000) was used for the interaction test of the aptamer-aptamer homodimeric and aptamer-antibody heterodimeric binding. The 5’-biotin-modified DNA aptamers for LCN2 (LCN2_apta2 and LCN2_apta4) were immobilized by passing 500 nM aptamers for 10 min at 10 μL per min on an SA chip. The SA chip was pre-activated with 1 mL of HBS-EP buffer (GE Healthcare, UK) at a flow rate of 10 μL per min for 10 min. The SA chip was pre-activated with 1 mL of the running buffer at a flow rate of 10 μL per min for 10 min. Then, the 5′-biotinylated aptamer sequence was immobilized on the chip as recommended by the manufacturer’s manual (**Supporting information** Figure S4). To monitor the interaction using the BIAcore instrument, all procedures were automatically implemented to create repetitive cycles of sample injection (90 μL injection samples, at a flow rate of 10 μL per min) and regeneration (1 M NaCl, 50 mM NaOH), according to the instruction guidelines. The binding interaction was analyzed by subtracting the response units of the blank flow cell from the response of the selected DNA for the flow cell (BIA evaluation program, version 4.1)^36^.

### Analysis for the binding orientation of LCN2-bound aptamer by dot blotting analysis

As a test for the preliminary characterization of each sandwich matched pair, dot blotting analysis was performed. To investigate the binding orientation of aptamer toward LCN2, 9 selected candidate aptamers (LCN2_apta1 to LCN2_apta9, 742 pmol) were incubated with LCN2 (74.2 pmol) for 1 h at 4 °C in separated reaction tubes. Then, 1 μL of each aptamer-LCN2 complex was spotted onto a Hybond-P PVDF membrane (GE Healthcare, Uppsala, Sweden). All samples were allowed to adsorb completely for 3–5 min. For the assay signal controls, antibody diluted solutions were made, and then 1 μL of each solution was spotted on the membrane (C3; primary antibody diluted 1:5 to 0.1 μg μL^−1^ and C4; secondary antibody diluted 1:4 to 0.1 μg μL^−1^). The membranes were placed in a square reaction chamber (2 cm x 10 cm) and then blocked with PBS-T buffer containing 5% nonfat dry milk for 1 h at room temperature. Next, dot blotting analysis was performed using an anti-LCN2 monoclonal antibody (Abcam, Cambridge, UK) as the primary antibody and anti-mouse IgG peroxidase conjugates (Sigma-Aldrich, Missouri, USA) as the secondary antibody. The membranes were washed three times for 10 min each time using washing buffer. Signals were detected by chemiluminescence with an enhanced chemiluminescence (ECL) detection system (GE Healthcare, Uppsala, Sweden)^37^. The signal intensity of each dot was corrected using ImageJ (National Institutes of Health). The signal of the background area immediately surrounding the dot was collected, averaged, and then subtracted from the assay signal of the dot. The analysis experiments were repeated three times.

### Specific and quantitative detection of LCN2 in the aptamer-based sandwich assay

Next, 5’-end amine-modified LCN2_apta4 (Capture_Aptamer) and HRP-labeled LCN2_apta2 (Reporter_Aptamer) molecules were synthesized (Bioneer, Dae Jeon, Korea). The following procedure was performed. (1) A DNA-binding 96-well plate (Corning Costar, Cambridge, MA, USA) was incubated with 1 μM of LCN2_apta4 for 1 h. (2) After blocking the well with PBS buffer containing 0.5% BSA, 100 pmol of protein samples (BSA, GST, HSA, AFP, and LCN2) was added to each well, and the plate was incubated for 1 h. (3) After non-binding molecules were washed away with PBS buffer, 1 μM of horseradish peroxidase (HRP)-labeled LCN2_apta2 was applied to each well for 30 min. In detail, LCN2_apta4 (Capture_Aptamer) was modified with an amino group at the 5’end so that it could be covalently coupled to the DNA-BIND™ plate that was coated with a layer of reactive N-oxysuccinimide esters (referred as NOS groups). Then, 5’-amine modified LCN2_apta4 was dissolved in PBS buffer at a concentration 1 μM (1 pmol μL^−1^); next, 100 μL per well was applied to the DNA-BIND plate. The plate was incubated for 1 h and washed three times with washing buffer, PBS-T (PBS, pH 7.0, 0.1% Tween 20) to remove uncoupled DNA. After extensive washing with PBS buffer, protein samples were added. The TMB solution (BioLegend, San Diego, USA) was subsequently incubated for detection. All steps were performed for 1 h at room temperature, and the reaction wells were washed three times with washing buffer for 10 min. The absorbance of each well was determined using a microplate reader set to 450 nm (**Supporting Information** Table S1). For the quantitative detection, LCN2 was added at concentrations ranging from 0–500 ng mL^−1^ and incubated for 1 h. The remaining procedures were the same as those described above.

### Detection of LCN2 in serum samples using the aptamer-based sandwich assay

After immobilizing the Capture_Aptamer (1 μM of LCN2_apta4), all serum samples were incubated for 1 h at 4 °C. Unbound materials were removed by washing with distilled water. The remaining procedures were the same as those described above. To compare with commercial ELISA kit performance, the overall assay was performed according to the manufacturer’s instructions (Abnova, Taiwan, China). For the quantitative ELISA application, all serum samples were diluted 30-fold in series with PBS buffer. Signals were measured by absorbance at 450 nm in a microplate reader.

### Statistical analysis

Statistical analyses were performed using the R Foundation for statistical computing software. Statistical differences were determined by the Student *t* –test. Results are expressed as the mean ± SEM. P-values > 0.05 were considered as not significantly different in the analytic comparison.

## Results

### ***In vitro*** selection of high-affinity DNA aptamer for LCN2

**Figure 1:**
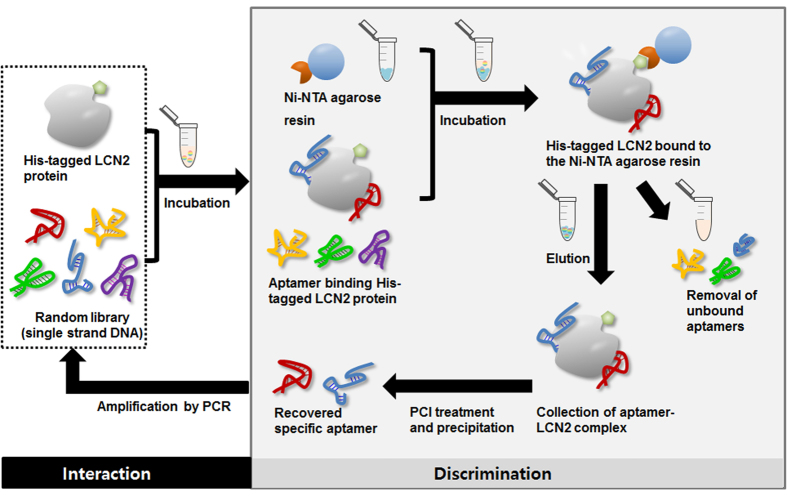
LCN2-SELEX (Systematic Evolution of Ligands by Exponential enrichment) process. The SELEX process starts with a very large library of DNA. After incubation with the random DNA library, the discrimination steps are performed to isolate aptamers that can specifically bind to LCN2. After a washing step to remove the unbound species, aptamer candidates are eluted and amplified by PCR. The amplified DNA is used to conduct another selection round. All parts of this figure were drawn by the authors K-A. L. and J-Y. A.

LCN2 was actively explored as a potential biomarker for HCC. The initial goal of the study was to investigate the capacity of the aptamers to serve as a diagnostic detection probe for LCN2 in a sandwich assay platform. The initial complex library, as a DNA aptamer, was evolved for LCN2 using the typical SELEX procedure. Figure 1 illustrates the screening and amplification of ssDNA aptamers bound to his-tagged human LCN2 recombinant protein over 10 selection rounds. The typical selection process involves target hybridization and discrimination between high-affinity binders and non-reactive or low-affinity binders, which is followed by PCR amplification. Finally, the enriched DNA aptamer species are cloned and identified as potential aptamers. After 10 rounds of SELEX, NanoDrop spectrophotometer analysis was performed to monitor and assess the recovery efficiency (percentage of bound DNA (%)) of aptamer pools to LCN2. The fact that binding did not appear to be present in Ni-NTA agarose resin (negative round, N.R) suggested that binding was specific for LCN2. (**Supporting Information** Figure S1 and Table S1).

**Figure 2:**
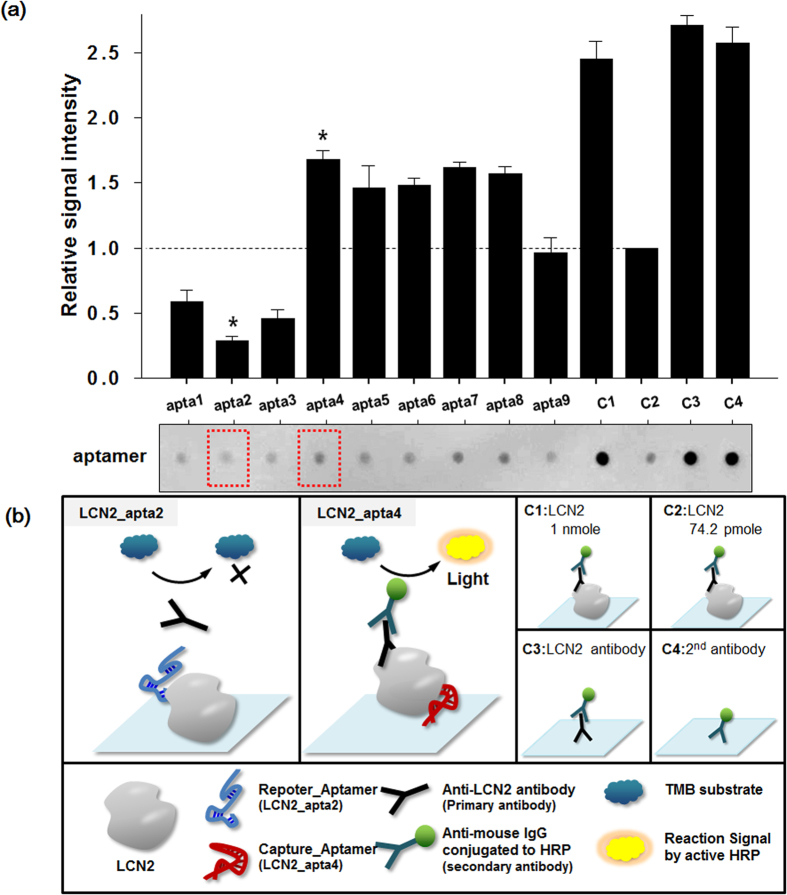
Dot blotting analysis for binding site confirmation. Dot blotting was performed to define the geometric orientations of the selected aptamers. (**a**) LCN2 (74.2 pmol) with 9 selected aptamers (LCN2_Apta1 to LCN2_Apta9, 742 pmol) and controls (C1 to C4) were dotted onto the Hybond-P PVDF membrane. After incubation with the HRP-conjugated anti-LCN2 polyclonal antibody, an assay image was taken using an ECL assay protocol. The plotted signal intensities were calculated using ImageJ software and normalized to C2 (LCN2 74.2 pmol). The schematic epitope binding of two aptamers (LCN2_apta2 and LCN2_apta4) is illustrated in (**b**). All parts of this figure were drawn by the authors K-A. L. and J-Y. A.

The specific ssDNA ligands bound in each round were monitored by post-SELEX analysis of the eluted samples. The eluted ssDNA bound from the 7^th^, 8^th^, and 9^th^ rounds was normalized to the same concentration and reintroduced to LCN2 for post-SELEX incubation. The recovered DNA samples from the 7^th^, 8^th^, and 9^th^ rounds were amplified using an optimized RT-PCR cycle, and the fluorescence signal was monitored with a MiniOpticon^TM^ real-time PCR fluorescence signal detection system (Bio-Rad, USA). Each PCR reaction was independently performed in triplicate, and data were analyzed to obtain the average C(t) values (Fig. 2). The samples from 8^th^ round of SELEX showed a lower C(t), C(t) = 14.15, compared to the values of other samples, C(t) = 15.20 for the 7^th^ round and 16.00 for the 9^th^ round, indicating higher levels of the DNA aptamer for LCN2 (**Supporting Information** Figure S2). The 8^th^ round was finally selected as the optimal enrichment round. We then cloned the PCR products from round 8 and arbitrarily sequenced 42 clones. Based on the SPR affinity test and sequence alignment using ClustalX software, we found that 42 aptamer candidates could be classified into 4 distinct groups. Nine aptamer candidates (named LCN2_apta1 to LCN2_apta9) with high affinity were finally considered for further experiments (Table 1 **and Supporting Information** Table S2).

### K_*D*_ determination by SPR affinity analysis

For the binding affinity analysis of the aptamers, a SPR experiment was subsequently performed for LCN2, and the specificity was calculated using BIA evaluation software. The binding affinities of the selected aptamers for LCN2 were evaluated by measuring the dissociation constant (*K*_*D*_) via SPR data. We used the CM5 chip (GE Healthcare, Sweden), a carboxymethylated 3D dextran matrix, which is a general purpose chip for the immobilization of a wide range of ligands, from small organic molecules to proteins, nucleic acids and carbohydrates^38^. The CM5 Chip that was immobilized with the LCN2 was investigated comparatively in terms of aptamer affinity (K_*D*_) using the BIAcore 3000 (GE Healthcare, Sweden).

The *K*_*D*_ of LCN2-bound aptamers has values in the range from 6.47 × 10^−13^–7.02 × 10^−9^ M (Table 1). Most of the aptamers that we isolated have *K*_*D*_ values ranging from pico- to nanomolar affinity. This high affinity may be due to factors such as the following: (1) efficient separation of target binding and nonbinding in the discrimination step, (2) appropriate binding conditions (buffer, temperature, and time) and (3) LCN2 target property. Further studies are needed to select a sandwich complex pair toward LCN2 in the assay platform.

### Aptamer pair by dot-blotting analysis

Dot-blotting analysis was carefully designed to select the best available aptamer pair for the sandwich complex. Interestingly, when LCN2 binding ssDNA aptamers were applied to a dot-blotting test, antibody interference on the epitope site was clearly observed. LCN2 protein mixtures with each ssDNA aptamer (LCN2_apta1 to LCN2_apta9) were separately blotted onto the PVDF membrane and assayed with anti-LCN2 monoclonal and HRP-conjugated secondary IgG antibodies in parallel. The LCN2 protein (Control 1 (C1), LCN2 (1 nmol) and Control 2 (C2), LCN2 74.2 pmol), primary antibody for LCN2 (Control 3 (C3), mouse anti-LCN2 antibody) and secondary antibodies (Control 4 (C4) and HRP-conjugated anti-mouse IgG) were used as positive controls and signal indicators. All signals collected by dot blotting were normalized to the corresponding signal for control C2 (LCN2 74.2 pmol). We observed that although the signal for LCN2_apta1-3 was reduced (relative signal intensity of LCN2_apta2: 0.28), LCN2_apta4-8 did not result in a signal-density reduction and had increased binding intensity (relative signal intensity of LCN2_apta4: 1.68) (Fig. 2a**, red-dotted boxes and see Supporting Information** Table S2). The signal reduction in the LCN2_apta2 spot may have been caused by the competitive inhibition of anti-LCN2 monoclonal antibody at the same functional epitope site, while LCN2_apta4 can react with LCN2 at an allosteric site, which may affect the enhancement of the LCN2 protein stability on the PVDF membrane (Fig. 2b). Most convincing is perhaps the fact that aptamers are basically oligonucleotides, and they have an electronegative charge, providing enhanced blotting strength on the membrane and open access to the antibody binding motif on the membrane. Precise orientation by apta4 as a flexible anchor may improve the binding signal.

No cross-reactivity between the aptamers (C) was observed. The substrate background (A, NOS functional substrate; B, adding a reporter aptamer; D, LCN2 and reporter aptamer complex, lacking a capture aptamer) was also regarded as invalid. As noted, we could conclude that LCN2_apta4 did not interfere with the binding of anti-LCN2 antibodies because there was no competition for antibody binding. LCN2_apta2 and LCN2_apta4 had different geometric orientations relative to LCN2 so they would not interfere with each other, as revealed by SPR (**Supporting Information** Figure S4). Therefore, it is emphasized that LCN2_apta2 and LCN2_apta4 can act in a sandwich complex, without binding competition, as a matched pair for determining LCN2.

### Characteristics of the aptamer sandwich pair for detecting LCN2

**Figure 3:**
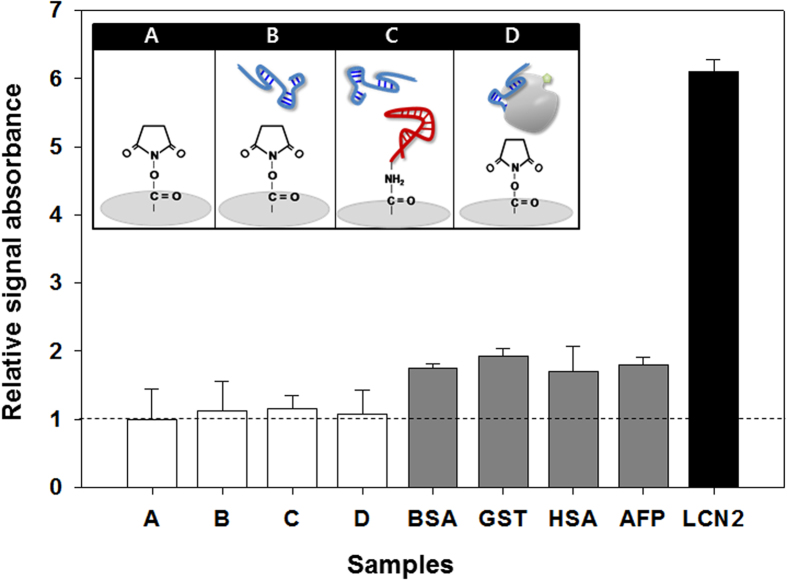
Specificity validation of the aptamer-based sandwich assay for LCN2. Nonspecific protein (gray columns), BSA, GST, HAS and AFP were prepared as described in the materials and methods section, and their signals were compared with LCN2 binding activity (black column). LCN2_Apta2 and LCN2_Apta4 were used and are highlighted with blue and red, respectively. The substrate background (**A**), NOS functional substrate; (**B**), adding a Reporter_Aptamer; and D, LCN2 and reporter aptamer complex, lacking capture aptamer) was examined. The cross-reactivity between aptamers (**C**) was also tested, as illustrated in the upper control boxes. The signal intensity was plotted relative to the NOS-group area on each well in a microplate (seeing the dot line at 1, A). The column heights represent the mean values, and error bars indicate the range of triplicate measurements. All parts of this figure were drawn by the authors K-A. L. and J-Y. A.

The aptamer-based sandwich assay has also been investigated for evaluating LCN2 using “Capture_Aptamer” and “Reporter_Aptamer” in further detail. The major advantage of the sandwich assay as a common laboratory technique is that it remains in expanded formats with analytic modifications that allow for multiple target-based outputs, a low affinity sensitive signal read-out and flexibility in terms of the assay development. A conceptual aptamer-based sandwich assay is based on research evidence that aptamers can provide a high specificity and affinity for a wide variety of targets^30^,^35^,^39^,^40^,^41^. LCN2 recognition in the aptamer-based sandwich assay was successfully performed using a matched aptamer pair, LCN2_apta2 and LCN2_apta4. LCN2_apta4 was considered a Capture_Aptamer because the SPR binding affinity tests revealed that LCN2_apta4 has a higher affinity at a *K*_*D*_ value of 6.09 × 10^−11^ M than that of LCN2_apta2 (*K*_*D*_ value of 2.24 × 10^−12^  M) (Table 1). After 1 μM of 5’ amine-modified LCN2_apta4 was covalently immobilized onto the wells of a 96-well microplate, the LCN2 binding signal was efficiently read using Reporter_Aptamer (1 μM, LCN2_apta2). The binding specificity, cross-reactivity between the capture and reporter aptamers, and background signal noise were analyzed using both specific and nonspecific proteins (**Supporting Information** Table S4). Figure 3 reveals that a distinctive signal was only observed by adding LCN2, not controls (A to D), nonspecific proteins (BSA, GST and HSA) or potential HCC biomarkers (AFP). This implies that the aptamer-based sandwich assay was responsible for the significant and specific binding to LCN2. In agreement with the dot blotting (Fig. 2) and SPR data (**Supporting Information** Figure S4), the capture and reporter aptamers did not react with each other (Fig. 3, **C in upper control box**).

### Quantitative approach for LCN2 with the aptamer-based sandwich assay

**Figure 4:**
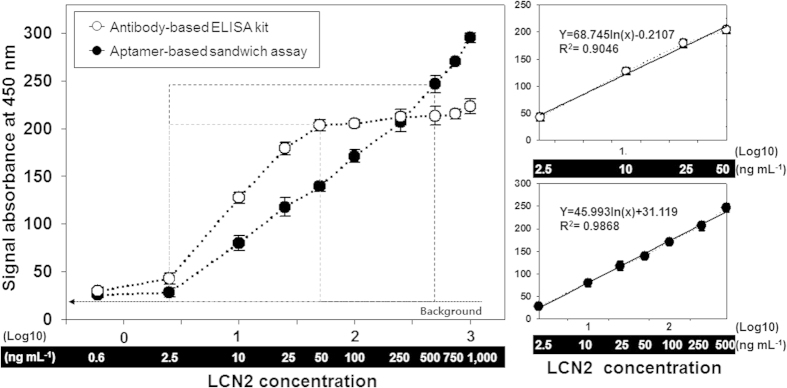
Broad linear regression of the aptamer-linked sandwich assay. A series of samples containing LCN2 (0.6, 2.5, 10, 25, 50, 100, 250, 500, 750 and 1,000 ng mL^−1^) was prepared and assayed with both aptamer and antibody-based sandwich (ELISA kit) platforms. The background is defined as the average signal from the LCN2 absence (0 ng mL^−1^), and the error bars represent the standard deviation. The concentration range of 50–500 ng mL^−1^ indicates a critical detection area of the aptamer assay for LCN2 (serological LCN2 value, see Table 2). The part highlighted with a gray box in the graph (2.5 ~50 ng mL^−1^) indicates a linear dynamic range of ELISA with the equation, **Y** **=** **68.745ln(x)-0.2107** (**R^2^** **=** **0.90).** The result of the aptamer sandwich construction shows that LCN2 was sensitively detected in the linear dynamic range of 2.5 ~ 500 ng mL^−1^ with the equation **Y** **=** **45.993ln(x)** **+** **31.119** (**R^2^** **=** **0.99).**

We set up the sandwich assay for the quantitative detection of LCN2 in 96-well microplates. For the binding assays, various concentrations of LCN2 (0.6, 2.5, 10, 25, 50, 100, 250, 500, 750 and 1,000 ng mL^−1^) were prepared and assayed with both aptamer- and antibody-based sandwich (ELISA kit) platforms. LCN2_apta was applied as a Capture_Aptamer (1 μM) and LCN2 was probed with Reporter_Aptamer (LCN2_apta2; 1 μM). The LCN2 levels were successfully detected. As the LCN2 concentration increased, the amount of Reporter_Aptamer also increased, leading to a higher measured response. Figure 4 shows the reliable and sensitive quantitation of LCN2 with a linear dynamic range of 2.5–500 ng mL^−1^ (**Y** **=** **45.993ln(x)** **+** **31.119** (**R^2^** **=** **0.99)**) and a limit of detection of 0.6 ng mL^−1^. This quantitation is sufficient to cover the serological LCN2 value in a serum sample (Fig. 4 and Table 2). The present approach can be critically applied for detecting biomarkers within the low and medium-abundance ranges of the protein concentration (ng mL^−1^ to μg mL^−1^, **see** Table 3).

We further compared our findings with the LCN2 results obtained from a commercially available antibody-based ELISA kit (Abnova, Taiwan). In Fig. 4, the part highlighted with a light-gray box compares the binding curves at increasing LCN2 concentrations. ELISA has a relatively narrow linear dynamic range of < 1 order of magnitude (2.5–50 ng mL^−1^**; Y** **=** **68.745ln(x)-0.2107** (**R^2^** **=** **0.90)**). The narrow dynamic range of the assay makes ELISA extremely sensitive for biomarker detection at the low abundant level (less than femto- to attomole levels)^42^. However, it requires several serial sample dilutions to obtain reliable concentrations, which is a time-consuming and labor-intensive process, limiting its clinical application. These results indicate that despite the novelty of the antibody-based ELISA in terms of the highly sensitive biomarker measurement, it may not be useful for calculating the actual biomarker levels within the nano- to microgram concentration ranges in patient serum.

### Clinical application of the aptamer-based sandwich assay

**Figure 5:**
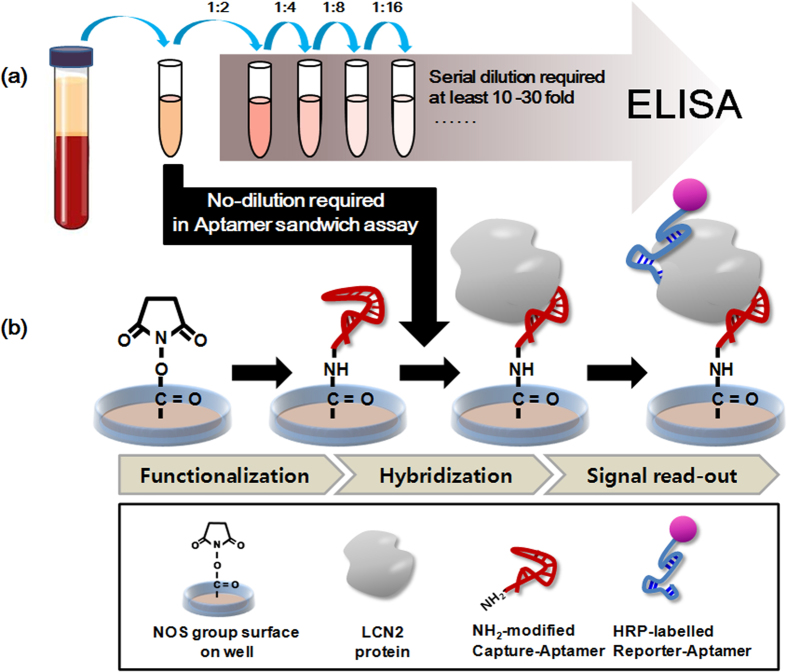
Schematic illustration of the aptamer-based sandwich assay. (**a**) Sample preparations for the use of the current antibody-based ELISA kit. (**b**) Aptamer-based sandwich assay platform consisting of the following three major factors: the functionalized surface by capture_aptamer (LCN2_apta4), hybridization, and signal read-out by HRP-labeled reporter_aptamer (LCN2_apta2), which enables the device to be run on straight serum, and there is no need for sample dilution. All parts of this figure were drawn by the authors K-A. L. and J-Y. A.

For the clinical approach, 8 serum samples from 4 normal individuals and 4 clinically relevant HCC patients were tested (Clinical scales: 8 (5 men and 3 women), aged between 30 to 78 years). As illustrated in Fig. 5, the aptamer sandwich assay consists of functionalization, hybridization and a signal read-out. The level of LCN2 in the serum samples was quantitatively measured with the aptamer-based sandwich assay, and all outputs from the assay were compared with the ELISA antibody performance. All samples for antibody-based ELISA were 30-fold diluted with PBS buffer solutions as described in the materials and methods section. For the data processing, the observed assay values were then multiplied by the dilution factor. Table 2 shows the quantitative responses of the aptamer assay. These results are very consistent with the current antibody-based ELISA kit performance and no sample dilution was required. Taken together, the aptamer approach is very reliable and can be used as a simple alternative to the conventional method, for both lab-scale prepared and patient samples. It is important that there was no statistically significant difference in the analytic comparison of the LCN2 concentrations between the aptamer-based assay and ELISA (p > 0.05).

## Discussion

The proposed aptamer-based sandwich assay platform is a more straightforward approach for detecting biomarkers in serum than the current ELISA assay. The approach is governed by the following three major factors: (1) the specific aptamer selection, (2) the evidence of an allosteric arrangement of the capture- and reporter-aptamer pair, and (3) the feasibility of a wide variety of serum disease markers. The SELEX process showed that an increasing number of oligonucleotides led to an apparent aptamer-enrichment for LCN2, while the aptamer binding signal was not observed in either the non-specific targets (GST, BSA and HSA) or potential HCC biomarkers (AFP) during the selection processes (Fig. 3**, Supporting Information** Figures S1 and S4). The selected aptamers, as capture- and reporter aptamers, were geometrically assessed based on dot blotting and antibody competition analysis, as shown in the dot blotting analysis (Fig. 2). As the binding epitope of a monoclonal antibody is known, antibody interference is indirect evidence of the ability of the two aptamer components to recognize their target in the different epitope sites. This confirmed that the aptamers specifically responded to the target LCN2 in the sandwich assay complex without cross-reactivity. In addition, LCN2 in clinical samples was quantitatively measured, and the results were comparable with those of the current antibody-based ELISA kit performance. We concluded that the proposed approach is applicable for the quantification of broad-scale abundance biomarkers without any extended dilution processes (Fig. 4 and 5).

To date, few studies have evaluated the biomarkers for multipurpose applications, such as early detection, disease monitoring, and prognosis^29^,^43^,^44^,^45^,^46^. The widespread use of the current assay platform may be limited by the specific purpose of each disease marker. Although highly sensitive ELISA assays are very useful for ultra-low abundant biomarkers in terms of early-stage diagnosis^27^,^47^,^48^,^49^, they are not suitable for prolonged disease monitoring or predicting subsequent disease progression because of the elevated serum levels over normal ranges. Table 3 lists the broad-spectrum biomarkers that have been shown to exist with clinical values in the patient serum. Low-medium abundance markers, such as PSA (4 ng mL^−1^), AFP (20 ng mL^−1^), SAA (34.1 ng mL^−1^), GPC3 (100 ng mL^−1^), and CRP (1 μg mL^−1^), have drawn attention as prognostic biomarkers for disease progression^50^. Their presence in the serum was significantly elevated over the normal serum levels as disease-associated responses in patients, and there was a degree of elevation from 5- up to 500, in the level of several hundred nanograms to a few micrograms per milliliter^17^. Therefore, accurate measurements over a wide range of serum levels are now essential to evaluating and predicting disease progression. Our platform can sufficiently quantify these prognostic biomarkers, ranging from ng mL^−1^ to μg mL^−1^, in patient serum.

Over the last decade, substantial research has centered on the development of assay platforms for detecting disease biomarkers with highly sensitive and high-resolution real-time analysis^4^,^6^,^7^,^8^,^9^,^51^. The demand for POCT diagnostic assays performed close to the patient has recently been increasing^52^,^53^,^54^. The aptamer sandwich assay can potentially fulfill the growing requirements for such a diagnostic assay. Studies with aptamers in lateral flow devices and disposable biosensors have demonstrated the stability and reliability of the assay with whole blood or patient serum^26^,^55^,^56^,^57^. The POCT set-up generally needs to be easy to handle, simple to manipulate and capable of rapid data processing in a single device, even when used by an untrained personnel^57^,^58^,^59^,^60^. The developed assay platform provides a simple and rapid method for detecting LCN2 in a serial order. The assay consists of functionalization, hybridization, and signal read-out, without requiring dilution. Therefore, this approach can be adapted in various diagnostic assays that focus on clinical approaches, such as POCT, lab on a chip (LOC), lateral flow (LF) devices and biosensor systems.

## Additional Information

**How to cite this article**: Lee, K.-A. *et al.* Aptamer-based Sandwich Assay and its Clinical Outlooks for Detecting Lipocalin-2 in Hepatocellular Carcinoma (HCC). *Sci. Rep.* **5**, 10897; doi: 10.1038/srep10897 (2015).

## Supplementary Material

Supplementary Information

## Figures and Tables

**Table 1:** Sequence data of the selected LCN2-binding aptamers and measurement of the binding ability between aptamers and LCN2.

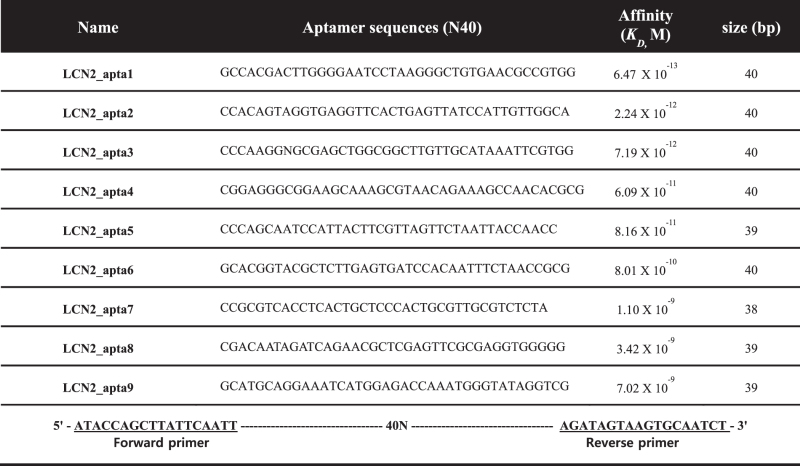

**Table 2:** LCN2 detection in serum. Eight samples from 4 HCC patients and 4 healthy individuals were directly applied to the aptamer-based assay well and then incubated with reporter aptamer. For comparison with the ELISA kit performance, serum samples were 30-fold serial diluted, and experiments were performed according to the manufacturer’s instructions. Statistical analyses were performed using the R Foundation statistical computing software. Statistical values were determined by Student’s *t*-test. Results are expressed as the mean ± SEM. P > 0.05 indicates that there was no significant difference in the analytic comparison between the ELISA kit and aptamer-based assay performance.

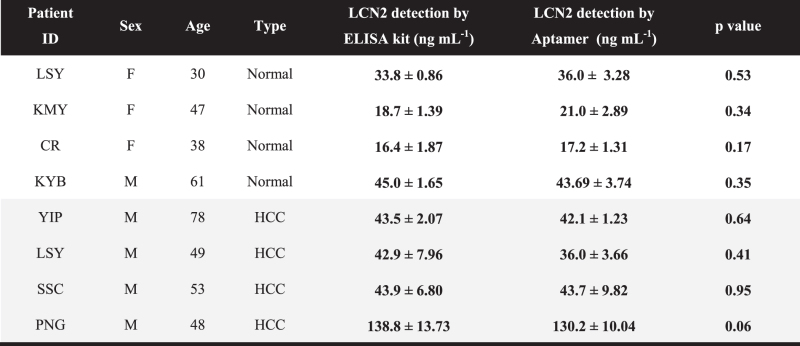

**Table 3:** Broad biomarker spectrum associated with the cancer diagnosis and prognosis.