# Supplementary material for: Aptamer-based Sandwich Assay and its Clinical Outlooks for Detecting Lipocalin-2 in Hepatocellular Carcinoma (HCC)
Source: Sci Rep. 2015 Jun 3;5:10897. doi: 10.1038/srep10897 (PMC4454046; doi:10.1038/srep10897)
Supplement: Supplementary Information [file srep10897-s1.doc]

**Supporting Information**

Title Aptamer-based Sandwich Assay and Its Clinical Outlooks for Detecting Lipocalin-2 in Hepatocellular Carcinoma (HCC)

Kyeong-Ah Lee^1^, Ji-Young Ahn^1^, Sang-Hee Lee^1^, Simranjeet Singh Sekhon^1^, Dae-Ghon Kim^2^, Jiho Min^3^ and Yang-Hoon Kim^1^,*

^1^Department of Microbiology, Chungbuk National University, 1 Chungdae-Ro, Seowon-Gu, Cheongju 362-763, South Korea

^2^Division of Gastroenterology and Hepatology, Research Institute of Clinical Medicine, Department of Internal Medicine, Chonbuk National University, Medical School and Hospital, Jeonju, 561-756, South Korea

^3^Graduate School of Semiconductor and Chemical Engineering, Chonbuk National University, 567 Baekje-daero, Deokjin-Gu, Jeonju, 561-756, South Korea

Keywords: Aptamer-based Sandwich Assay, ELISA, DNA Aptamer, LCN2, HCC, POCT

**Abbreviations**

Lipocalin2 (LCN2); Hepatocellular carcinoma (HCC); Surface Plasmon Resonance (SPR); Point of Care Testing (POCT); Hepatitis B virus (HBV); Hepatitis C virus (HBC); α-fetoprotein (AFP); des-γ-carboxy prothrombin (DCP); protein induced by vitamin K absence or antagonist (PIVKA-II); neutrophil gelatinase-associated lipocalin (NGAL); Systematic Evolution of Ligands by Exponential Enrichment (SELEX); Glutathione S-transferase (GST); Bovine Serum Albumin (BSA); Human Serum Albumin (HSA); 3-dimentional (3-D); real time-polymerase chain reaction (RT-PCR); negative round (N.R); polyvinylidene difluoride (PVDF)

**Figures and Tables**

**Figure S1. Measurement of eluted aptamers during the SELEX processes.** The ssDNA of each round was obtained from each selection round. Negative round (N.R) was performed after round 6 to prevent enrichment of LCN2 nonspecific binding aptamer. ssDNA pools eluted from round 8 indicated high concentration selection, and are pointed with a black arrow.

The higher appearance of initial two rounds seemed to be caused by nonspefic binding.

**Figure S2. Post-SELEX and RT-PCR analysis.** The specific ssDNA ligands bound in each round was monitored by post-SELEX analysis of the eluted sample. The eluted ssDNA bound from 7^th^, 8^th^, and 9^th^ round was normalized and reintroduced to LCN2 for post-SELEX incubating. The recovered DNA samples from 7^th^, 8^th^, and 9^th^ round were amplified using an optimized RT-PCR cycle and the fluorescence signal was monitored by MiniOpticon^TM^ Real-time PCR fluorescence signal detection system (Bio-Rad, USA). PCR experiment was independently triplicated and data was analyzed to obtain the average C(t) values.

**Figure S3. The binding affinity test by SPR.** The isolated all aptamers (42 clones) against LCN-2 were analyzed by Surface Plasmon Resonance using a Biacore 3000 Instrument (GE Healthcare, Sweden) at 24°C. We used the CM5 chip (GE Healthcare, Sweden), a carboxymethylated 3D dextran matrix, which is a general purpose chip for immobilization of a wide range of ligands such as proteins, nucleic acids and carbohydrates. Immobilization of LCN2 on a CM5 chip (GE Healthcare, Uppsala, Sweden) was carried out according to the standard protocol of the amine-coupling method (BIAapplication Handbook, Biacore® AB). All the sequence information and affinity data were listed in Supporting Information Table S2.

**Figure S4. Cross-reactivity test.** 5’**-**Biotinylated LCN2_apta2 and apta 4 were immobilized on Streptavidin SPR sensor chip. As targets, LCN2 protein, anti-LCN2 monoclonal antibody, anti-mouse secondary antibody, potential HCC biomarker (AFP) and counter aptamers were used. (a) LCN2_apta 2 immobilized SA chip test and (b) LCN2_apta4 immobilized SA chip test. Except LCN2 target (①), there was no binding activity between aptamer and targets.

**Table S1. Percentage of bound DNA aptamer in SELEX**

**Table S2. Sequence analysis and K*_D_* determination of aptamers by SPR**

**Table S3. Dot blotting analysis for aptamer binding**

**Table S4. Specificity validation of aptamer-based sandwich assay for LCN2**

**Figure S1**

**
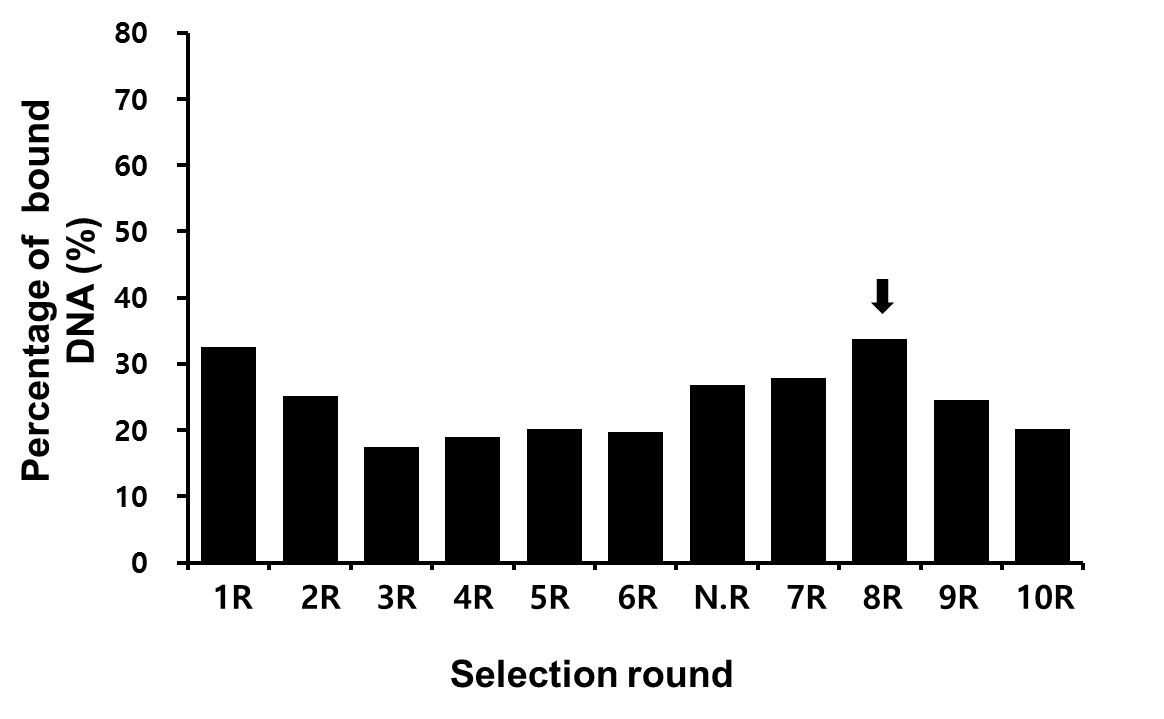
**

**Figure S2**


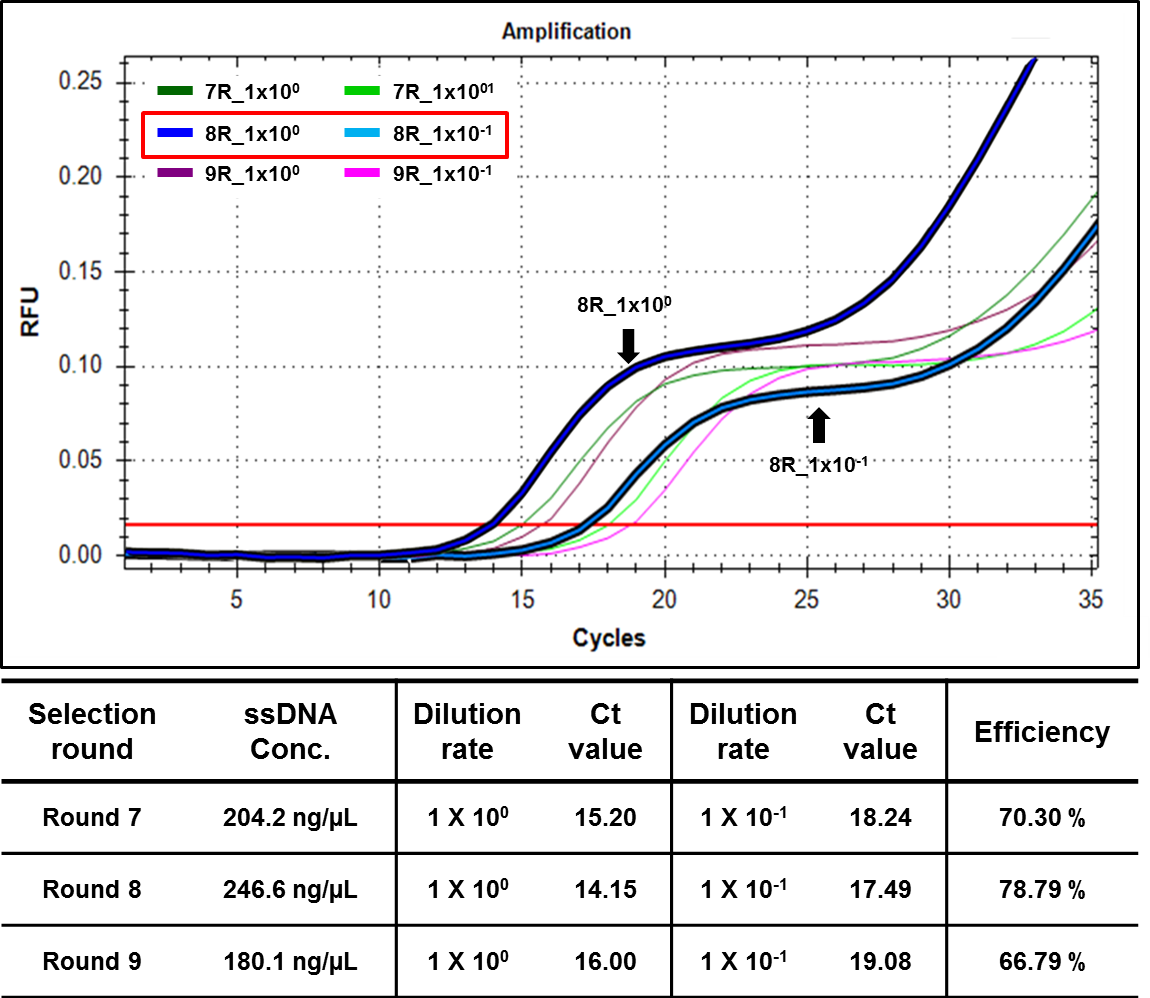


**Figure S3**

**
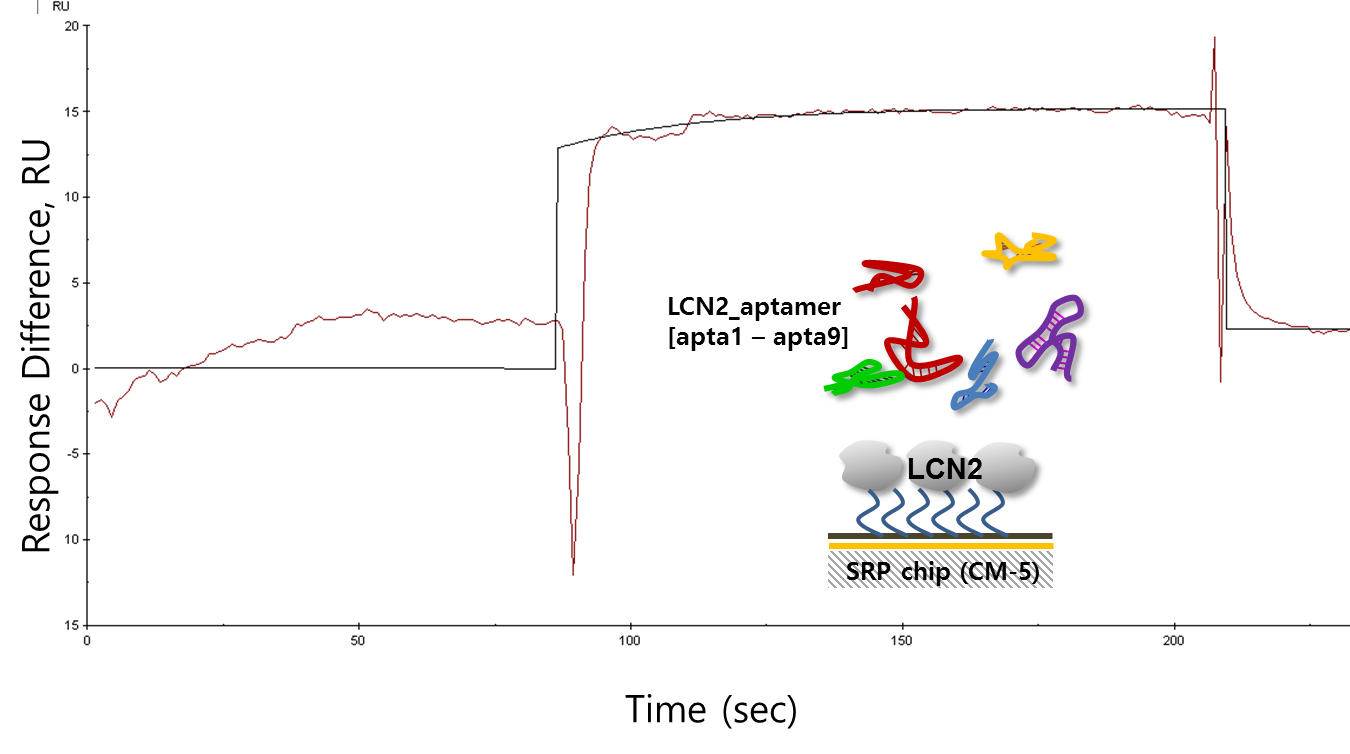
**

**Figure S4(a)**

**
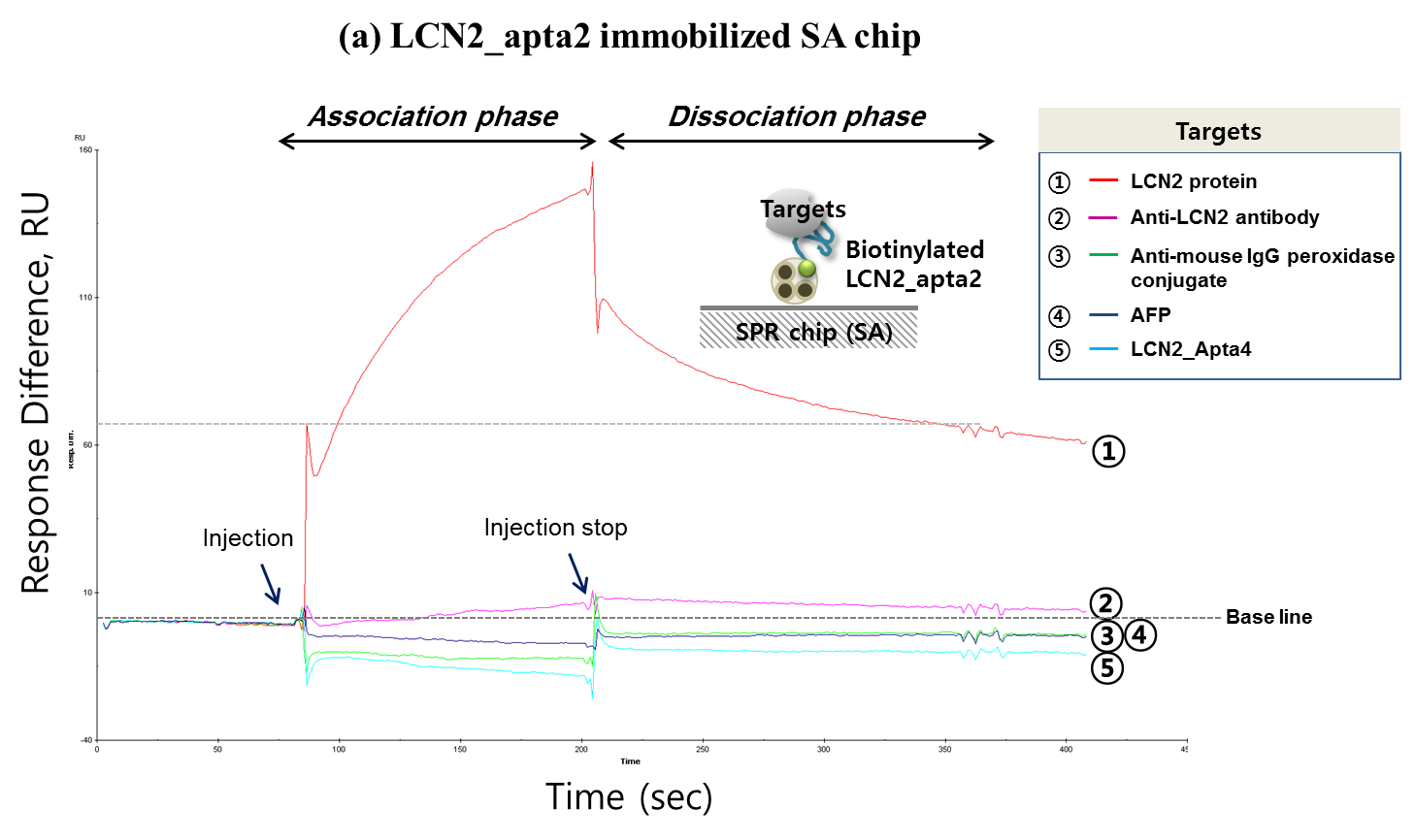
**

**Figure S4(b)**

**
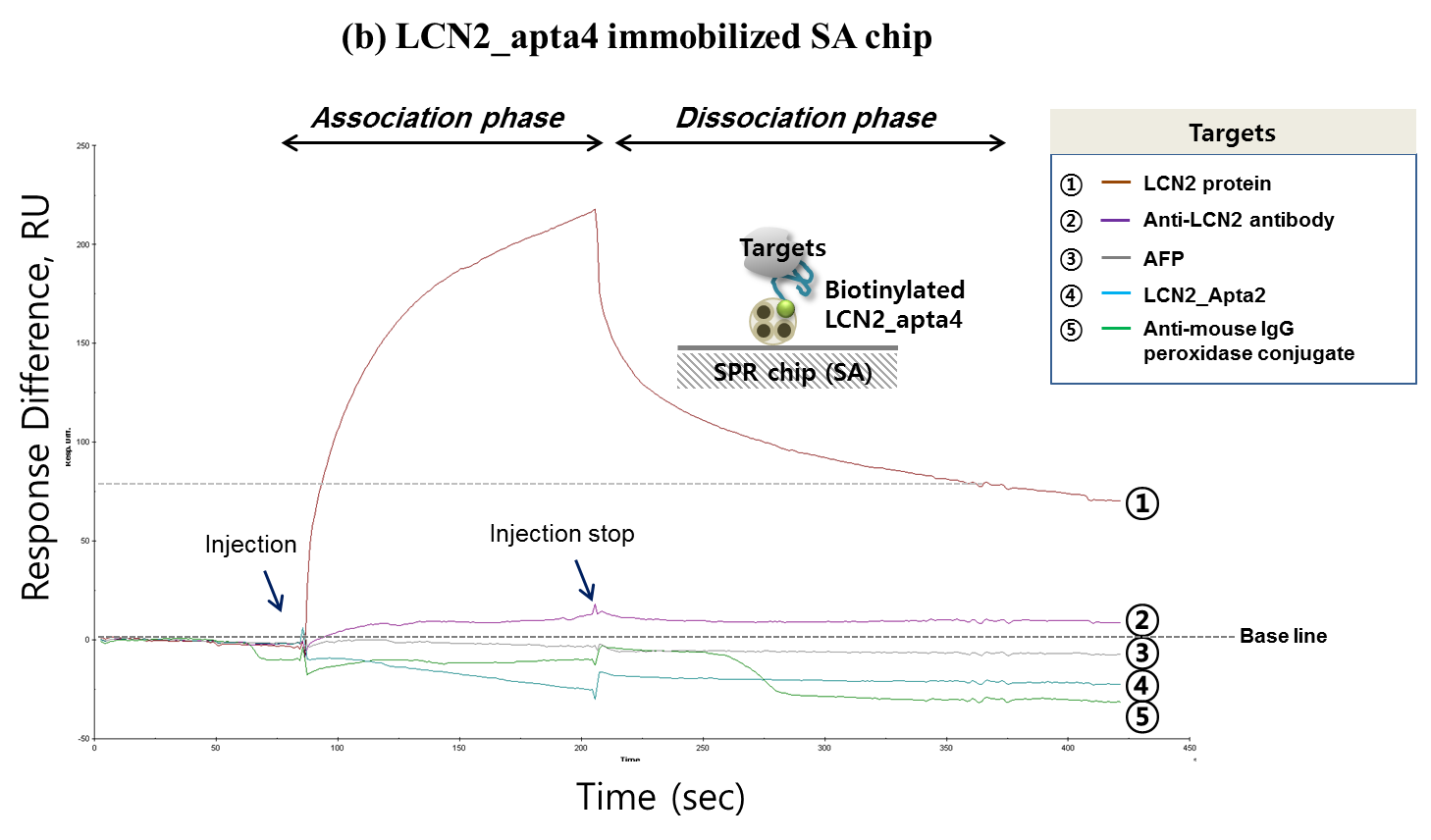
**

**Table S1. Percentage of bound DNA aptamer in SELEX**

| **SELEX**  **round** | **ssDNA mixed with LCN2** | **Eluted ssDNA from LCN2** | **Binding Percentage** |
| --- | --- | --- | --- |
|  | **742pmole (18,300 ng)** | **(ng)** | **(%)** |
| **1 Round** | 18,300 | 5950 | 32.5 |
| **2 Round** | 18,300 | 4612.5 | 25.2 |
| **3 Round** | 18,300 | 3182.5 | 17.4 |
| **4 Round** | 18,300 | 3470 | 19.0 |
| **5 Round** | 18,300 | 3687.5 | 20.2 |
| **6 Round** | 18,300 | 3607.5 | 19.7 |
| **N.R** | 18,300 | 4905 | 26.8 |
| **7 Round** | 18,300 | 5105 | 27.9 |
| **8 Round** | 18,300 | 6165 | 33.7 |
| **9 Round** | 18,300 | 4502.5 | 24.6 |
| **10 Round** | 18,300 | 3685 | 20.1 |

**Table S2. Sequence analysis and K*_D_* determination of aptamers by SPR**

| **Group** | **Name** | **Aptamer sequences (N40)** | **Affinity**  **(*K_D_,* M)** | **Random**  **sequences size (bp)** | | **The number**  **of clone** |
| --- | --- | --- | --- | --- | --- | --- |
| I | **LCN2_apta1** | GCCACGACTTGGGGAATCCTAAGGGCTGTGAACGCCGTGG | 6.47 X 10^-13^ | | 40 | 1 |
|  | **LCN2_apta2** | CCACAGTAGGTGAGGTTCACTGAGTTATCCATTGTTGGCA | 2.24 X 10^-12^ | | 40 | 3 |
|  | **LCN2_apta3** | CCCAAGGGCGAGCTGGCGGCTTGTTGCATAAATTCGTGG | 7.19 X 10^-12^ | | 39 | 1 |
|  | **LCN2_apta4** | CGGAGGGCGGAAGCAAAGCGTAACAGAAAGCCAACACGCG | 6.09 X 10^-11^ | | 40 | 6 |
|  | **LCN2_apta5** | CCCAGCAATCCATTACTTCGTTAGTTCTAATTACCAACC | 8.16 X 10^-11^ | | 39 | 1 |
|  | **LCN2_apta6** | GCACGGTACGCTCTTGAGTGATCCACAATTTCTAACCGCG | 8.01 X 10^-10^ | | 40 | 1 |
|  | **LCN2_apta7** | CCGCGTCACCTCACTGCTCCCACTGCGTTGCGTCTCTA | 1.10 X 10^-9^ | | 38 | 1 |
|  | **LCN2_apta8** | CGACAATAGATCAGAACGCTCGAGTTCGCGAGGTGGGGG | 3.42 X 10^-9^ | | 39 | 1 |
|  | **LCN2_apta9** | GCATGCAGGAAATCATGGAGACCAAATGGGTATAGGTCG | 7.02 X 10^-9^ | | 39 | 1 |
| II | **LCN2_apta20** | CCACAAACGGTACTGGACAGTTCTTATATGCTTACATTTG | 1.09 X 10^-8^ | | 40 | 1 |
|  | **LCN2_apta27** | GCGATAATCTTGGTTTTCTTTTCAGTTTCTTTAGGGCGGG | 1.39 X 10^-8^ | | 40 | 1 |
|  | **LCN2_apta19** | CGACAAAGGAGCTACGTCTACATAGCGTGGTGAGTGCTTG | 6.66 X 10^-8^ | | 40 | 1 |
|  | **LCN2_apta35** | GCATGCAGGAAATCATGGAGACCAAATGGGTATAGGTCG | 8.91 X 10^-8^ | | 39 | 1 |
|  | **LCN2_apta12** | CAGGGAAAGGTTACGCGACACATTAGTTTTGAGTCCGTTC | 8.45 X 10^-8^ | | 40 | 1 |
|  | **LCN2_apta30** | GCACGGGTAAGAAAACGGGGAACTTGAAAGTAGTGCTGTG | 8.64 X 10^-8^ | | 40 | 1 |
|  | **LCN2_apta14** | CGGCCAGATGCTTTGTCGCGCTACAGTATTANCGTCCTGG | 8.93 X 10^-8^ | | 40 | 1 |
|  | **LCN2_apta10** | GGCAGTAGGGCGGGGGGGAGCATGGAGCACATTCTTGTCG | 9.99 X 10^-8^ | | 40 | 1 |
| III | **LCN2_apta26** | CCAACACCTTGGGTATCCTGTTATATTCACTTGTTGAGTA | 1.18 X 10^-7^ | | 40 | 1 |
|  | **LCN2_apta23** | CGTATCAGAATCGCGTGTCACCTTATCTGCCGTATGGGTG | 1.58 X 10^-7^ | | 40 | 1 |
|  | **LCN2_apta16** | CCGCGTCACCTCACTGCTCCCACCTGCGTTGCGTCTCTA | 2.20 X 10^-7^ | | 39 | 1 |
|  | **LCN2_apta29** | CCGTGACAAGTTGCAGGGGCTGTGCGGTTCTTTTTTTTTG | 5.65 X 10^-7^ | | 40 | 1 |
|  | **LCN2_apta21** | CCGGATTTTCGCACGGCTAGCGGGGAACCACTGTCTGGG | 8.72 X 10^-7^ | | 39 | 1 |
|  | **LCN2_apta31** | CCCGATCAGAACATCGGCTCTTATACCCGTTTTGGTCACG | 1.78 X 10^-6^ | | 40 | 1 |
|  | **LCN2_apta34** | CGGAGCGGTAGTTTGAGGAGGAAACAACCCGGCCTTGGAG | 2.87 X 10^-6^ | | 40 | 1 |
|  | **LCN2_apta18** | CCAGCAAGTAAAGGTTATTTACTAGTTAGGGGCTTCTTG | 3.38 X 10^-6^ | | 39 | 1 |
|  | **LCN2_apta22** | CCAACGAGGATGCCAAAGAAAGGTGAGGTGGTCCTAAGGG | 8.44 X 10^-6^ | | 40 | 1 |
|  | **LCN2_apta28** | ACCAACTCGTGAAACCCCGGGTGGTTACTTCCTTACTTGG | 2.16 X 10^-3^ | | 40 | 1 |
|  |  |  |  | |  |  |
| IV | **LCN2_apta11** | GCGATCGACGCCTGGAGAAGTTCCAGCATTAGCGTAAGTG | *Unbound* | | 40 | 1 |
|  | **LCN2_apta13** | CGCGGCCTAACTTTGGAAGTTCCTCAGAATACTCAGTTG | *Unbound* | | 39 | 1 |
|  | **LCN2_apta15** | CCACACCAGTCCTTGTTCGTAGATCACGGTTTCTTCAGGA | *Unbound* | | 40 | 1 |
|  | **LCN2_apta17** | CACGTGCGACAGGGGGATCTGGCTTTATCCCAGCGTGTTA | *Unbound* | | 40 | 1 |
|  | **LCN2_apta24** | GGGGAACTGAATTATCAGAGATTTTATTCACCTCGTTGGG | *Unbound* | | 40 | 1 |
|  | **LCN2_apta25** | GCCACTCGACATGCTTGCCTGTACACTAAAACACTATTC | *Unbound* | | 39 | 1 |
|  | **LCN2_apta32** | CGAGTCATTGTTTTGCAAAGGATTATGCGTTGTAACGGAG | *Unbound* | | 40 | 1 |
|  | **LCN2_apta33** | CACGACGATAGTGCAGTGGATACATTCGGCCCTTACCATG | *Unbound* | | 40 | 1 |
|  | **Total** |  |  | |  | **42** |

**Table S3. Dot blotting analysis for aptamer binding**

| **Aptamer** | **Observed signal intensity** | | **Relative signal intensity** | |
| --- | --- | --- | --- | --- |
|  | **Signal intensity** | **Standard deviation** | **Revised value** | **Standard deviation** |
| **LCN2_apta1** | 1333.698 | 351.3144 | 0.5862 | 0.0890 |
| **LCN2_apta2** | 651.92 | 158.3592 | 0.2865 | 0.0520 |
| **LCN2_apta3** | 1042.163 | 278.8347 | 0.4580 | 0.0713 |
| **LCN2_apta4** | 3822.891 | 507.555 | 1.6802 | 0.0672 |
| **LCN2_apta5** | 3320.406 | 229.0644 | 1.4593 | 0.1708 |
| **LCN2_apta6** | 3375.527 | 457.1815 | 1.4836 | 0.0507 |
| **LCN2_apta7** | 3680.941 | 551.6174 | 1.6178 | 0.0400 |
| **LCN2_apta8** | 3578.355 | 468.6037 | 1.5727 | 0.0530 |
| **LCN2_apta9** | 2193.548 | 523.5087 | 0.9641 | 0.1172 |
| **C1** | 5577.598 | 443.6256 | 2.4514 | 0.1400 |
| **C2** | 2275.305 | 498.7889 | 1 | - |
| **C3** | 6164.648 | 475.6599 | 2.7094 | 0.0800 |
| **C4** | 5860.698 | 384.2061 | 2.5758 | 0.1200 |

**Table S4. Specificity validation of aptamer-based sandwich assay for LCN2**

| **Sample** | **Signal absorbance (450 nm)** | | **Relative signal absorbance** | |
| --- | --- | --- | --- | --- |
|  | **Absorbance** | **SEM^*^** | **Revised value** | **SEM^*^** |
| A | 3.96068 | 1.7430 | 1 | 0.4400 |
| B | 4.48391 | 1.6986 | 1.1321 | 0.4288 |
| C | 4.6025 | 0.7310 | 1.1620 | 0.1845 |
| D | 4.2692 | 1.4195 | 1.0779 | 0.3584 |
| BSA | 6.9363 | 0.2714 | 1.7512 | 0.0685 |
| GST | 7.6257 | 0.4433 | 1.9253 | 0.1119 |
| HSA | 6.7254 | 1.4938 | 1.6980 | 0.3771 |
| AFP | 7.1199 | 0.4311 | 1.7976 | 0.1088 |
| LCN2 | 24.1930 | 0.6598 | 6.1082 | 0.1665 |

***SEM**: Standard Error of the Mean
